# Transcatheter Aortic Valve Replacement and Percutaneous Coronary Intervention After Ozaki Procedure in Alagille Syndrome

**DOI:** 10.1016/j.jaccas.2025.104148

**Published:** 2025-07-23

**Authors:** Benedikt Mayr, Nazan Puluca, Johannes Böhm, Christian Nöbauer, Salvatore Cassese, Michael Joner, Heribert Schunkert, Markus Krane, Hendrik Ruge

**Affiliations:** a Department of Cardiovascular Surgery, Institute Insure, German Heart Center Munich, School of Medicine and Health, Technical University of Munich, Munich, Germany; b Department of Cardiovascular Diseases, German Heart Center Munich, School of Medicine and Health, Technical University of Munich, Munich, Germany; c DZHK (German Center for Cardiovascular Research) – Partner Site Munich Heart Alliance, Munich, Germany

**Keywords:** aortic valve, percutaneous coronary intervention, valve repair

## Abstract

**Background:**

The Ozaki procedure is a novel therapeutic alternative to aortic valve replacement that involves excision of the native valve and replacement with neo-leaflets trimmed from the patient's pericardium.

**Case Summary:**

A 35-year-old man with Alagille syndrome and severe symptomatic stenosis of his neo-aortic valve was admitted 4 years after an Ozaki procedure. Given his high risk of redo surgery and nonsusceptibility to oral anticoagulation, valve-in-valve transcatheter aortic valve replacement (TAVR) with RESILIA tissue was performed. Despite preoperative imaging and good feasibility of TAVR after Ozaki, temporary obstruction of the right coronary artery occurred after TAVR, which was successfully resolved by ostial stent implantation.

**Discussion:**

Published reports on TAVR after Ozaki are sparse, especially in patients with rare multisystem disorders such as Alagille syndrome.

**Take-Home Message:**

TAVR after Ozaki in patients with Alagille syndrome can be successfully performed by a multidisciplinary heart team with special care regarding potential coronary occlusion.

## Graphical Abstract

**Figure:**
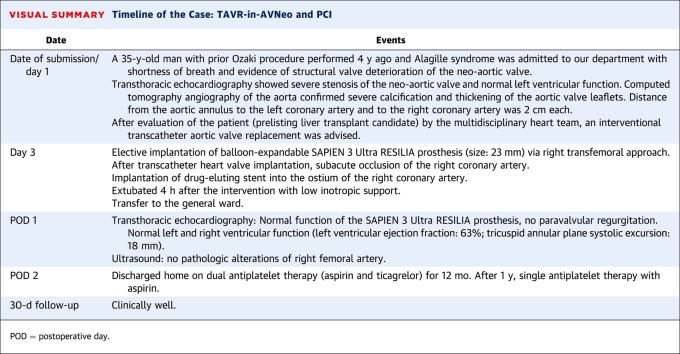

## History of Presentation

A 35-year-old man with Alagille syndrome and concomitant liver cirrhosis (Child-Pugh score: 7; Model for End-Stage Liver Disease score: 11) was admitted to our clinic owing to severe stenosis of the neo-aortic valve leaflets 4 years after aortic valve neocuspidization (AVNeo) using the Ozaki technique. In addition to cholestatic hepatic dysfunction, the patient had unilateral renal atrophy, splenomegaly, and significant stenosis of the celiac trunk and superior mesenteric artery. Symptoms included increasing shortness of breath during physical exercise (NYHA functional class II) and fatigue. Laboratory work-up revealed normal kidney function (serum creatinine: 0.74 mg/dL; urea: 25.3 mg/dL) and no alteration of the extrinsic and intrinsic coagulation system. However, impaired liver function (total bilirubin: 3.61 mg/dL; aspartate aminotransferase: 80.4 mU/L) was detected.

## Past Medical History

The patient had undergone AVNeo using autologous pericardium (neo-commissure: 1; size of neo-cusp: 25 mm for each) for severe symptomatic bicuspid (type 1) aortic valve stenosis (mean transvalvular gradient: 67 mm Hg; effective orifice area: 0.76 cm^2^) 4 years ago in our department. The patient attended routine follow-up in our department including annual transthoracic echocardiography (TTE) examination. At 2 years after surgery, an increase of the mean transvalvular gradient was observed (postoperative: 11 mm Hg; 1 year: 12 mm Hg; 2 years: 16 mm Hg) with slight thickening and sclerotic alteration of the left coronary cusp. At 3 years after AVNeo, additional thickening and impaired movement of the right coronary cusp was detected, and further increase of the mean transvalvular gradient to 33 mm Hg was recorded. Owing to possible thrombosis of the neo-aortic valve, the patient received the anticoagulation medication phenprocoumon for 3 months.

## Investigations

**Figure 1:**
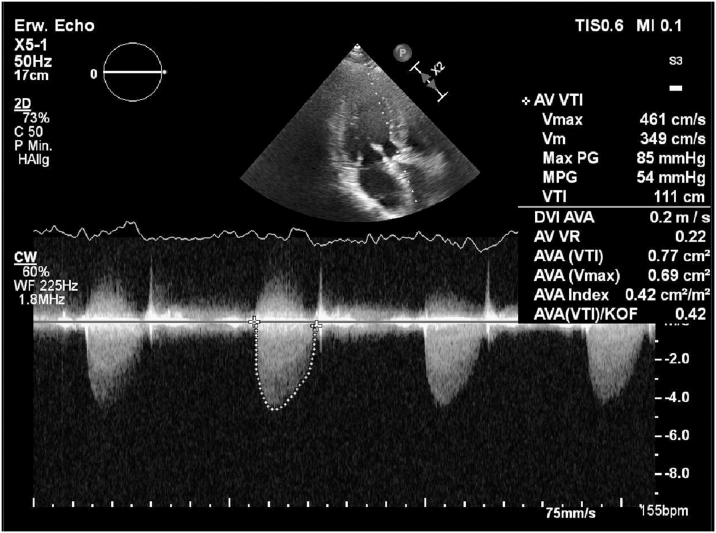
Measurement of the Severely Stenotic Neo-Aortic Valve by Transthoracic Echocardiography

**Figure 2:**
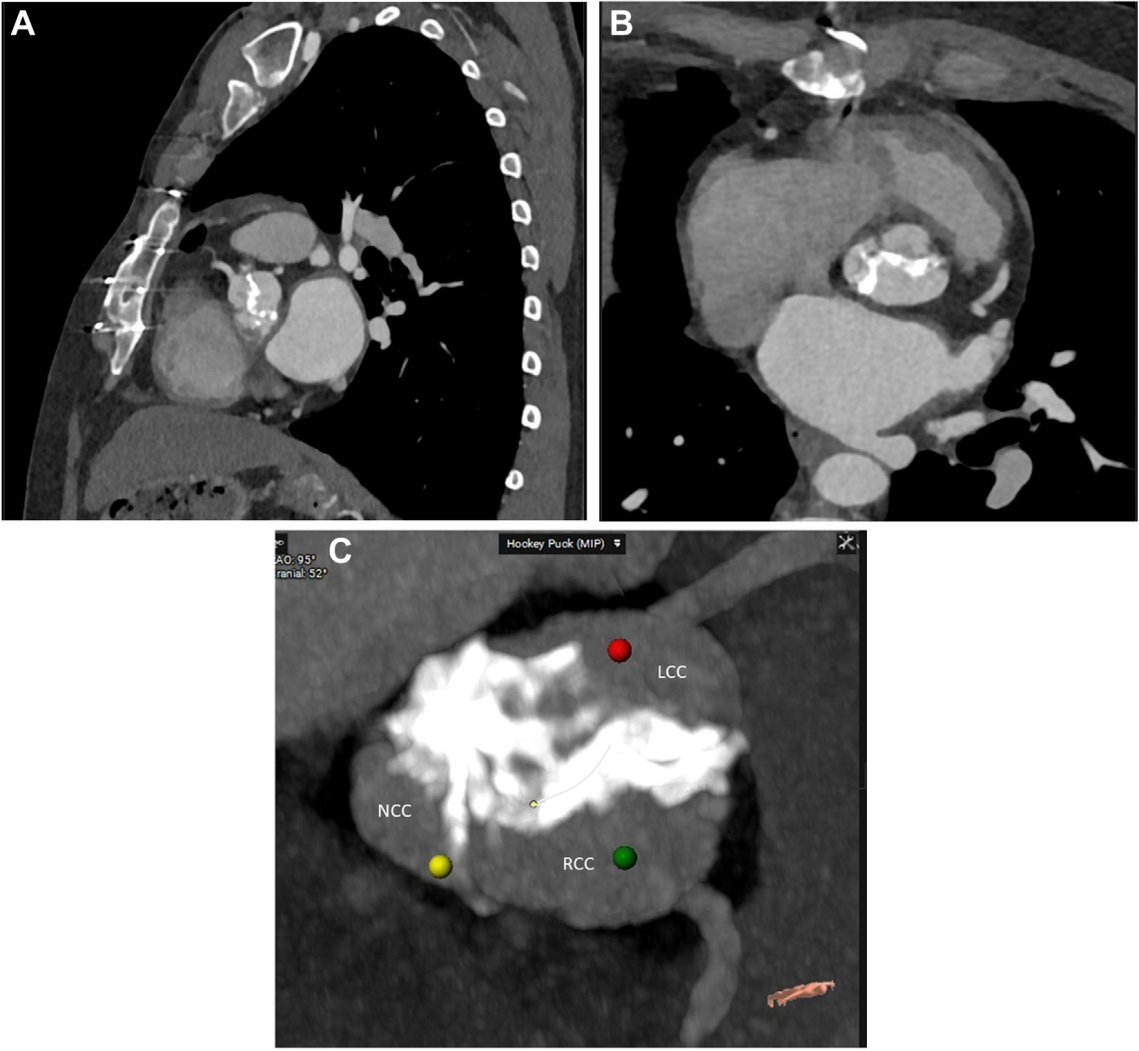
Computed Tomography Imaging of the Degenerated AVNeo Sagittal (A) and axial (B, C) computed tomography scan showing calcification and thickening of the left neo-valve leaflet. LCC = left coronary cusp; NCC = noncoronary cusp; RCC = right coronary cusp.

**Figure 3:**
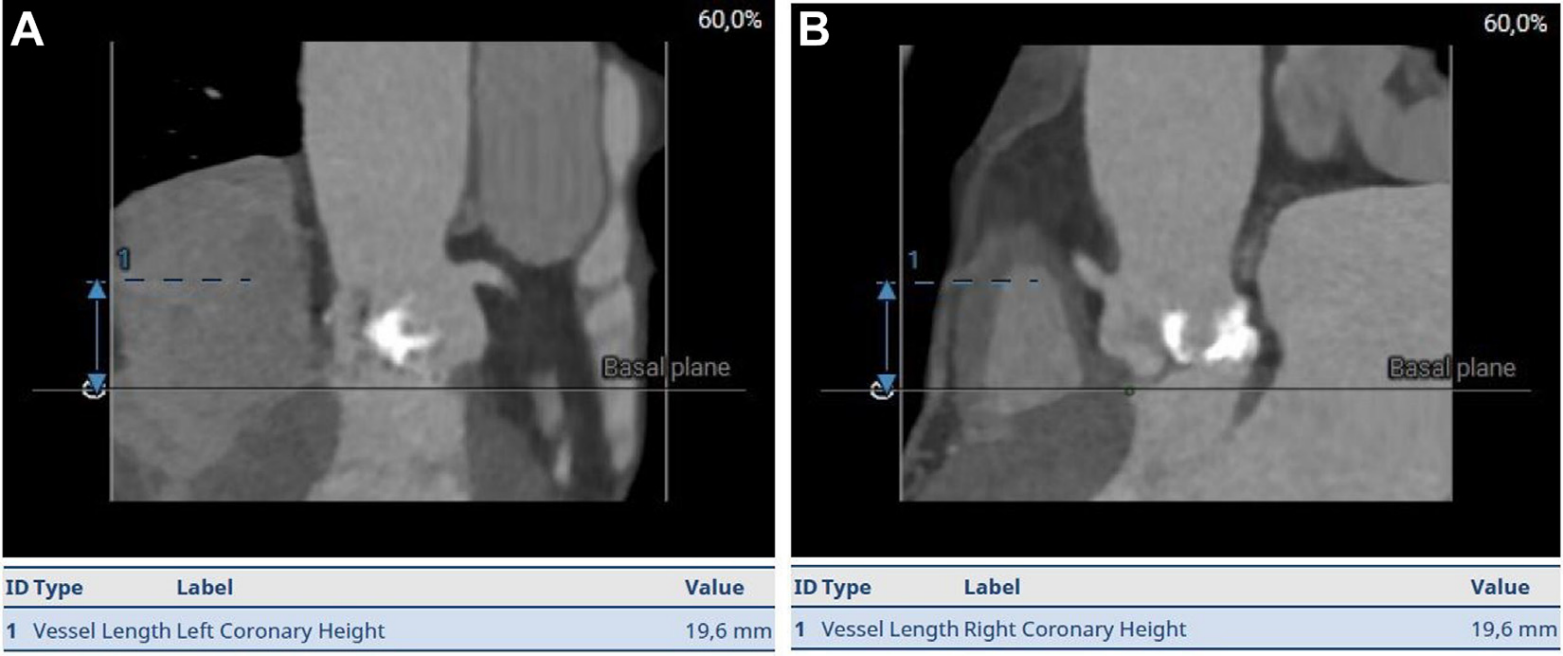
Height of the Coronary Ostia Relative to the Aortic Annular Plane Computed tomography measurement of distance from the aortic annulus to the left (A) and right (B) coronary artery.

Despite oral anticoagulation for 3 months, TTE showed persistent severe stenosis of the neo-aortic valve with decreased right coronary cusp and left coronary cusp leaflet mobility and additional mild regurgitation (maximum transvalvular gradient: 85 mm Hg; mean transvalvular gradient: 54 mm Hg; aortic flow velocity: 4.6 m/s; effective orifice area: 0.77 cm^2^) (Figure 1). Stenosis of the reconstructed aortic valve with thickened and calcified leaflets was confirmed by multidetector computed tomography (CT) (Figures 2A to 2C, Video 1). Aortic annulus area was 365 mm^2^ with a minimal and maximal diameter of 17 mm and 26 mm, respectively. The leaflet height and length of the right coronary neo-cusp was 13.4 mm and 14.2 mm, respectively (Supplemental Figure 1). The commissure height of the right to left coronary cusp was 13.9 mm, and the commissure height of the right to noncoronary cusp was 11.3 mm. The total leaflet calcium was 1,562 mm^3^ (Supplemental Figure 2). Sinus of Valsalva width was 20.7 × 19.2 mm (Supplemental Figure 3). Distance from the aortic annulus to the left coronary artery as well as to the right coronary artery measured 1.96 cm (Figures 3A and 3B). After evaluation of the patient (EuroSCORE II: 2.03%; Society of Thoracic Surgeons score: 1.5%) and the CT scan by the multidisciplinary heart team, an interventional approach with implantation of a balloon-expandable valve with RESILIA tissue (lower calcium binding) via transfemoral access as bridge to liver transplantation was advised.

## Management

**Figure 4:**
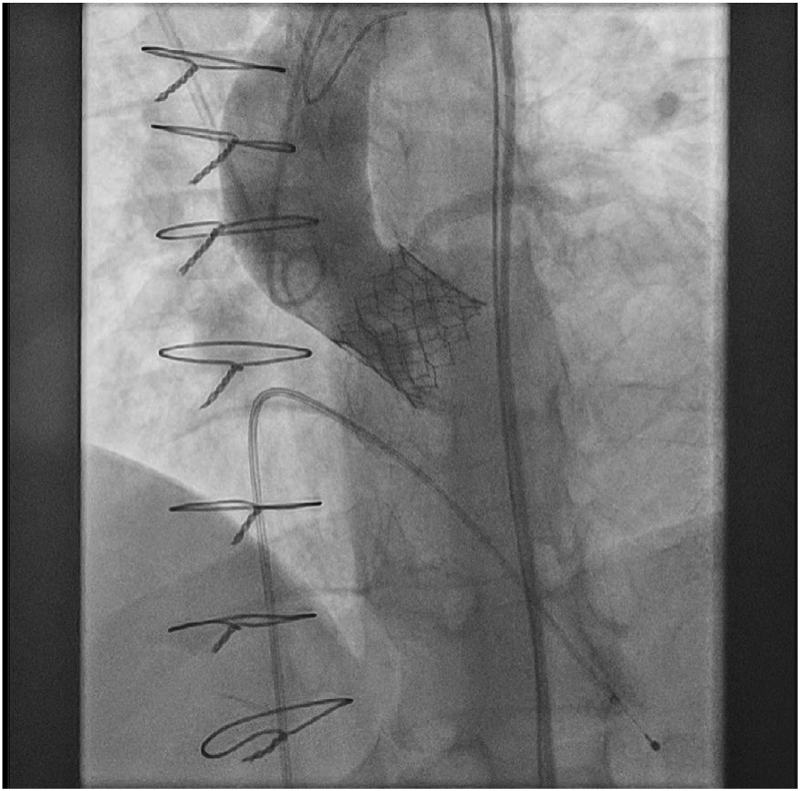
Aortography Intraprocedural aortography with deployed SAPIEN 3 Ultra RESILIA prosthesis and visualization of the left coronary artery. The right coronary artery is occluded.

**Figure 5:**
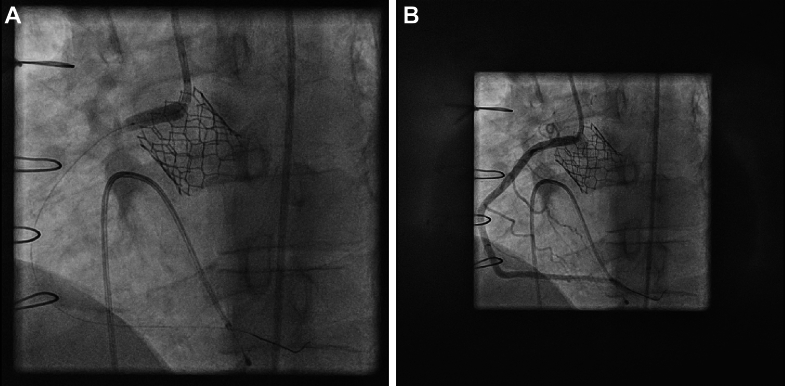
Percutaneous Coronary Intervention Aortography depicting drug-eluting stent implantation into the proximal right coronary artery (A) and unobstructed perfusion of the right coronary artery (B).

After transvenous placement of a temporary pacing wire in the right ventricle, retrograde passage of the aortic valve was achieved via percutaneous access of the right common femoral artery. Invasive aortic valve peak gradient measured 139 mm Hg. A balloon-expandable SAPIEN 3 Ultra RESILIA prosthesis (Edwards Lifesciences) (size: 23 mm) was advanced into the aortic annulus over an AMPLATZ extra-stiff guidewire (Cook Medical). Under rapid ventricular pacing and aortic root angiography, the transcatheter heart valve (THV) was deployed in the intended annular position. Aortic root angiography excluded a paravalvular leakage. However, right coronary artery perfusion was not confirmed (Figure 4). Electrocardiogram showed ST-segment elevations in leads II, III, and aVF. Hemodynamics remained stable. To secure airways, the patient underwent orotracheal intubation and mechanical ventilation. Incomplete occlusion of the right coronary ostium was confirmed by selective coronary angiography requiring immediate implantation of a 4.5 mm × 18 mm Resolute Onyx stent (Medtronic, Inc) (Figures 5A and 5B, Video 2).

## Outcome and Follow-Up

Postoperatively, the patient was hemodynamically stable on low-dose inotropic support and was extubated after 4 hours. On the day of the intervention, the patient was transferred to the normal ward. TTE confirmed intended THV performance (mean transvalvular gradient: 16 mm Hg, no paravalvular leakage) and normal left and right ventricular function. Two days after transcatheter aortic valve replacement (TAVR) after AVNeo (TAVR-in-AVNeo) and percutaneous coronary intervention, the patient was discharged from the hospital on dual antiplatelet therapy (aspirin and ticagrelor) for 12 months. After 1 year, single antiplatelet therapy with aspirin was advised. The patient is doing well clinically 6 months after the intervention, and TTE shows stable THV gradients.

## Discussion

**Figure 6:**
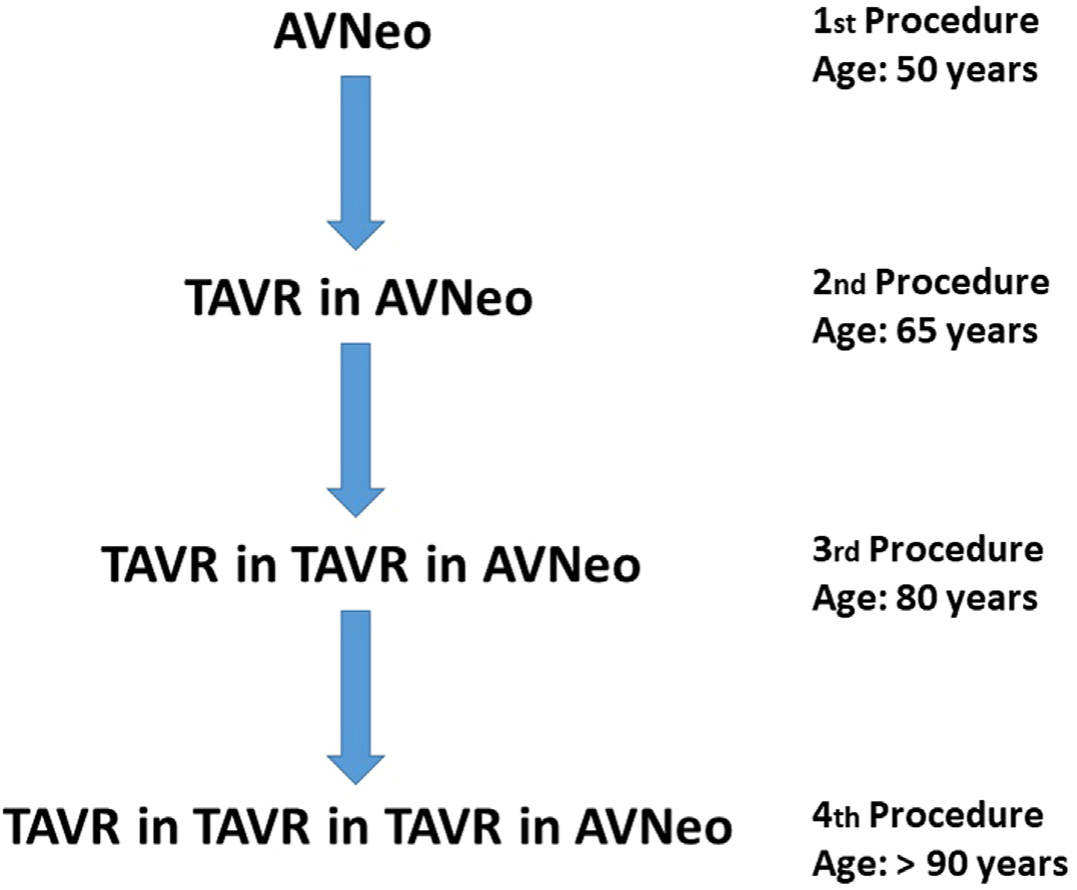
Interventional Treatment Timeline After AVNeo AVNeo = aortic valve neocuspidization; TAVR = transcatheter aortic valve replacement.

Alagille syndrome is an autosomal dominant multisystem disorder that involves hypoplasia of the intrahepatic bile ducts and cardiac and pulmonary vascular abnormalities.^1^^,^^2^ Transplantation of the liver is indicated in 21% to 33% of patients with Alagille syndrome, but eligibility is impacted by pulmonary artery hypoplasia and bicuspid aortic valve stenosis.^2^^,^^3^ Our patient was diagnosed with severe bicuspid aortic valve stenosis and fusion of the right and left coronary cusp (type 1). As the aortic annulus diameter was small (22 mm) and a mechanical prosthesis with mandatory oral anticoagulation was not an option for our patient with Alagille syndrome and compromised liver function, we performed AVNeo according to the Ozaki protocol. Another consideration for our treatment strategy was the fact that the patient's brother, who also had Alagille syndrome and aortic valve stenosis died as a result of a fatal hemorrhagic event after aortic valve replacement with a mechanical prosthesis at another institution. Despite various published reports on favorable mid-term results after AVNeo with low incidence of structural valve deterioration,^4^^,^^5^ severe structural valve deterioration was evident as soon as 3.6 years after AVNeo. Yet, no reports on a failing AVNeo in a patient with Alagille syndrome have been published, though the chronic hepatic inflammation and damage might have accelerated degenerative changes of the reconstructed valve leaflets. With progressive end-stage liver disease and scheduled listing for liver transplant, we opted for a catheter-based approach (TAVR-in-AVNeo) and implantation of a THV with RESILIA tissue due to a possible extended durability as bridge to transplantation. Aortic root dimensions and geometry seemed feasible for TAVR, and sclerosis of the neo-leaflets supported stable THV anchoring. Not foreseen in the CT evaluation, incomplete obstruction of the right coronary artery occurred requiring immediate right coronary ostial percutaneous coronary intervention. A possible reason for the coronary occlusion might have been the neo-leaflet length, despite the distance from the right coronary ostium to the aortic valve annulus measuring 1.96 cm. Although there is no cutoff established for valve-in-valve TAVR, there is general agreement to accept a cutoff of 1.2 cm distance as is the case for TAVR of the native aortic valve.^6^ In the light of potential coronary occlusion, it is important to consider that the Ozaki procedure almost doubles the coaptation surface of the neo-leaflets demonstrated in a three-dimensional CT modeling and in silico design by Macé et al.^7^ This technical detail, combined with a narrow aortic root diameter, an oval-shaped annulus, and asymmetric calcifications of the neo-leaflets, might increase the risk of an adverse coronary event, as seen in the present case. A possible technique for mitigation of coronary malperfusion during TAVR-in-AVNeo in patients with low coronary ostia is the reduction of leaflet height during the initial AVNeo procedure. This issue was addressed by Ozaki in 2021 with the introduction of a new template. Interventional techniques to prevent coronary obstruction include percutaneous coronary intervention with or without chimney stenting or leaflet laceration with transcatheter electrosurgery. Published reports on TAVR-in-AVNeo are still rare; however, Tada et al^8^ reported on successful TAVR-in-AVNeo with a SAPIEN 3 valve. With TAVR-in-AVNeo a valve-in-native annulus implantation can be performed, facilitating further valve-in-valve approaches and expanding the patient's possible interventional treatment time line^9^ (Figure 6).

## Conclusions

TAVR is a suitable approach for severe structural valve deterioration after AVNeo to avoid redo open heart surgery.

## Funding Support and Author Disclosures

Dr Krane is a physician proctor and a member of the medical advisory board for JOMDD, is a physician proctor for Peter Duschek, is a medical consultant for Evotec and Moderna, and has received speakers’ honoraria from Medtronic and Terumo. All other authors have reported that they have no relationships relevant to the contents of this paper to disclose.

**Take-Home Messages**

• Alagille syndrome is associated with congenital aortic valve stenosis and seems to lead to early degeneration of AVNeo.
• Sudden right coronary obstruction during TAVR-in-AVNeo is a rare but potentially fatal adverse event that can be resolved by urgent percutaneous coronary intervention.

## Appendix

Video 1 Computed tomography axial view of the full aortic valvular complex anatomy and valve calcification localization from the annulus.

Video 2 Transcatheter aortic valve replacement after aortic valve neocuspidization with SAPIEN 3 Ultra RESILIA prosthesis (size: 23 mm) and incomplete occlusion of the right coronary ostium with immediate implantation of a Resolute Onyx stent.
