# Supplementary material for: Transcatheter Aortic Valve Replacement and Percutaneous Coronary Intervention After Ozaki Procedure in Alagille Syndrome
Source: JACC Case Rep. 2025 Jul 23;30(20):104148. doi: 10.1016/j.jaccas.2025.104148 (PMC12441554; doi:10.1016/j.jaccas.2025.104148)

**Supplemental figure legends**

Supplemental figure 1: Computed tomography measurement of the leaflet height and length of the right coronary neo cusp

Supplemental figure 2: Computed tomography measurement of the leaflet calcium volume

Supplemental figure 3: Computed tomography measurement of the sinus of valsalva width

Supplemental figure 1


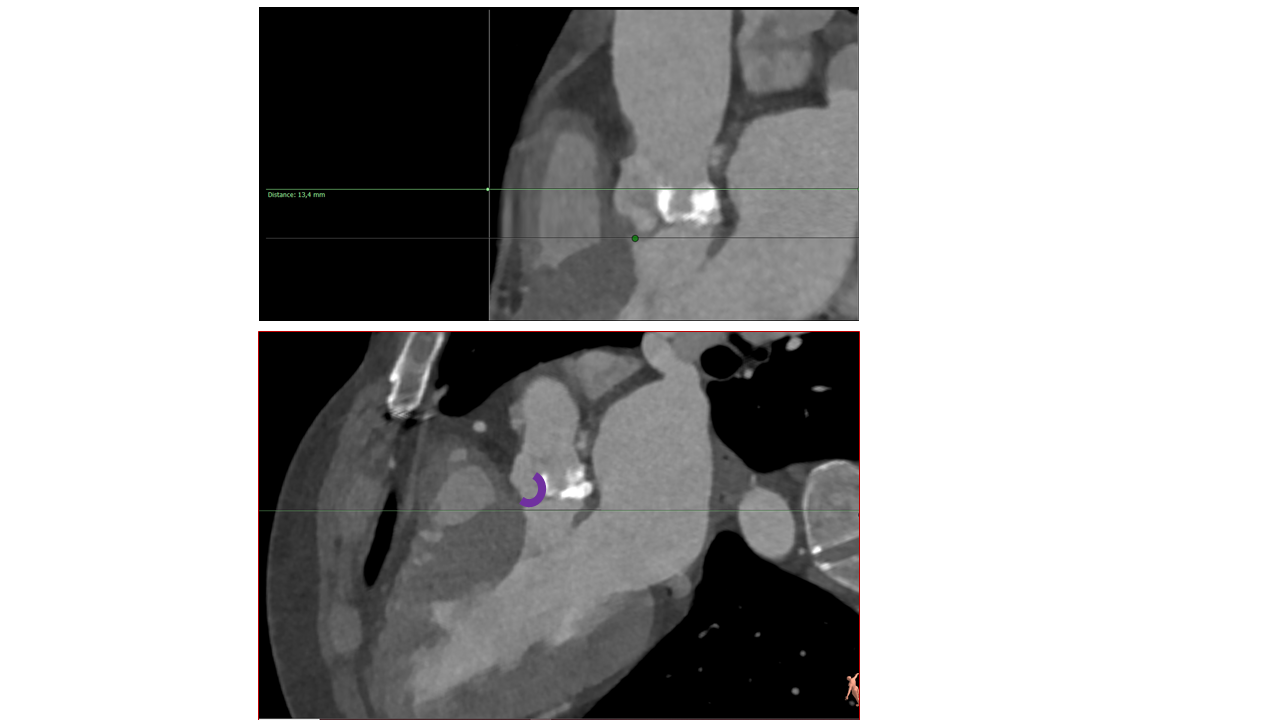


Supplemental figure 2


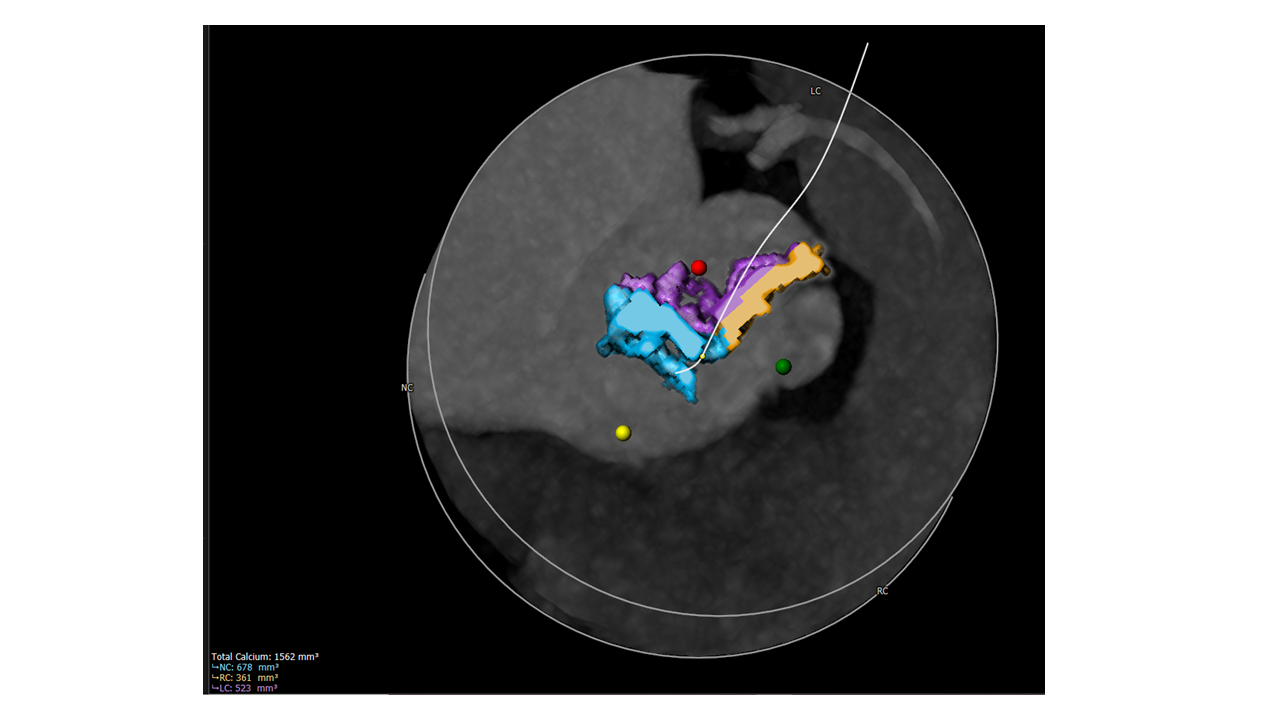


Supplemental figure 3


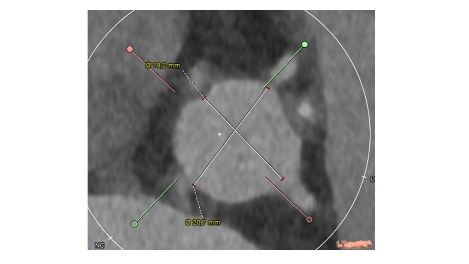

Supplement: Supplementary Figure 1-3 [file mmc3.docx]
